# Electric field bridging-effect in electrified microfibrils’ scaffolds

**DOI:** 10.3389/fbioe.2023.1264406

**Published:** 2023-10-25

**Authors:** Sara Fontana, Laura Caramazza, Paolo Marracino, Irene Cuenca Ortolá, Micol Colella, Noemi Dolciotti, Alessandra Paffi, Fernando Gisbert Roca, Sergiy Ivashchenko, Jorge Más Estellés, Claudia Consales, Marco Balucani, Francesca Apollonio, Micaela Liberti

**Affiliations:** ^1^ BioEM Lab, Department of Information Engineering, Electronics and Telecommunications (DIET), Sapienza University of Rome, Rome, Italy; ^2^ Center for Life Nano- & Neuro-Science, Fondazione Istituto Italiano di Tecnologia (IIT), Rome, Italy; ^3^ Rise Technology S.R.L, Rome, Italy; ^4^ Center for Biomaterials and Tissue Engineering, Universitat Politècnica de València, Valencia, Spain; ^5^ Division of Health Protection Technologies, ENEA-Italian National Agency for New Technologies, Energy and Sustainable Economic Development, Rome, Italy

**Keywords:** tissue engineering, biocompatible scaffold, microfibrils, electric stimulation, numerical modeling, dosimetry

## Abstract

**Introduction:** The use of biocompatible scaffolds combined with the implantation of neural stem cells, is increasingly being investigated to promote the regeneration of damaged neural tissue, for instance, after a Spinal Cord Injury (SCI). In particular, aligned Polylactic Acid (PLA) microfibrils’ scaffolds are capable of supporting cells, promoting their survival and guiding their differentiation in neural lineage to repair the lesion. Despite its biocompatible nature, PLA is an electrically insulating material and thus it could be detrimental for increasingly common scaffolds’ electric functionalization, aimed at accelerating the cellular processes. In this context, the European RISEUP project aims to combine high intense microseconds pulses and DC stimulation with neurogenesis, supported by a PLA microfibrils’ scaffold.

**Methods:** In this paper a numerical study on the effect of microfibrils’ scaffolds on the E-field distribution, in planar interdigitated electrodes, is presented. Realistic microfibrils’ 3D CAD models have been built to carry out a numerical dosimetry study, through Comsol Multiphysics software.

**Results:** Under a voltage of 10 V, microfibrils redistribute the E-field values focalizing the field streamlines in the spaces between the fibers, allowing the field to pass and reach maximum values up to 100 kV/m and values comparable with the bare electrodes’ device (without fibers).

**Discussion:** Globally the median E-field inside the scaffolded electrodes is the 90% of the nominal field, allowing an adequate cells’ exposure.

## 1 Introduction

In the last decades, a huge interest in tissue engineering (TE) has grown, which aims at finding innovative strategies to regenerate damaged biological tissues after diseases and injuries. The innovation of TE lies in the synergy of multidisciplinary approaches, based on highly advanced engineering and life sciences (Langer and Vacanti, 2016), such as stem cell biology, functional biocompatible scaffolds, nanotechnology, and three-dimensional (3D) bioprinting (Ramos and Moroni, 2020). In particular, bioprinting involves the assembly of biomaterials, bioactive molecules, and cells that are then manufactured to obtain engineered structures to substitute the damaged tissue and restore its functions (Ramos and Moroni, 2020). Such technology could represent an innovative strategy within regenerative medicine, alternative to transplants, mechanical devices, or surgical reconstruction. Among the wide application fields, it is possible to mention bone, cartilage, cardiac, pancreas, skin, and vascular tissue engineering (Castells-Sala et al., 2015). For instance, in the field of bone TE, several innovations have been introduced, thanks to the increasingly sophisticated 3D bioprinting technology proposed in the case of bone fractures or osteo-degenerative diseases. Nonetheless, given the challenge of patient-specific bone reconstruction, vascularization, and neuronal functionalization, even 4D-printed alternatives are explored. The aforementioned technology is capable of changing over time in shape, in order to adapt to the defect area environment, as a consequence of internal cell forces or external stimuli, and in order to allow the functionalization and maturation in time of the cells (Wan et al., 2020).

Furthermore, one of the most challenging applications of TE is the treatment of the central nervous system (CNS) and peripheral nervous system (PNS) injuries, which are unlikely to self-regenerate and could even cause permanent functional deficits (Boni et al., 2018), resulting in 6.8 million deaths each year (World Health Organization, 2007). CNS pathologies, such as neurodegenerative diseases and strokes, or PNS nerve injuries could cause neural connection impairment or inflammatory states, leading to cell death. Therefore, scientific research is focused on finding clinical solutions to reestablish the original neural pathways in the damaged tissue and restore the lost functionality. Implantable biomaterial scaffolds are increasingly employed for these purposes. They can be realized with natural polymers (such as cellulose, collagen, hyaluronic acid, and gelatin) to create a stable and nutritious environment to promote tissue regeneration (Schmidt and Leach, 2003; Wang et al., 2018). Nonetheless, in the last few decades, synthetic biocompatible polymers have been drawing more attention since they are cheaper and are characterized by a higher degradation time, which makes them suitable for human implantable devices; for these reasons, they are increasingly used to host and facilitate the growth of different cell types (i.e., stem cells), charged to migrate to the injury site and heal the tissue (Schmidt and Leach, 2003; Wang et al., 2018). Among the most frequently employed polymers, we possibly mention polylactide (PLA), polyglycolide (PGA), poly-l-lactide-co-glycolide (PLGA), and polycaprolactone (PCL) (Janouskova, 2018). Moreover, several research groups are focused on studying the proper scaffold’s biophysical characteristics and shape, including the addition of bioactive molecules, to promote the cell survival and proliferation and guide stem cell differentiation in the neuronal lineage (Wang et al., 2018).

Within these strategies, highly aligned fibrillar substrates have been deeply investigated to guide axonal growth in the direction of the fibers (Zhang et al., 2021). Specifically, the use of aligned nanofibers is useful to guide the dorsal root ganglion neurite growth and glial cell migration (Chow et al., 2007; Corey et al., 2007; Schnell et al., 2007; Gisbert Roca et al., 2020). Xie J et al. presented a conductive core-sheath PCL or PLA nanofibers’ scaffold coated with pyrrole (PPy) that showed improvement in neurite extension from cultured dorsal root ganglia (DRG) on uniaxially aligned nanofibers, compared with randomly oriented fibers’ scaffold (Xie et al., 2009; Gisbert Roca et al., 2020). Nevertheless, the scaffold’s dimension could affect the axonal growth, which is more favored by microfiber-based substrates instead of the nanofiber-based substrates (F. Gisbert Roca et al., 2020; Gisbert Roca et al., 2022). Outcomes of Yang and colleagues’ work pointed out that the scaffold highly supports neural stem cell (NSC) cultures, which elongated along the fibers, and improves the neurite outgrowth, thus demonstrating that the aligned PLA fibrous scaffold could be a potential cell carrier in neural tissue engineering (Yang et al., 2005). Furthermore, a huge interest in scaffolds’ electric functionalization is growing since exogenous electric stimulation significantly increases NSC proliferation, their differentiation into the neuronal lineage, and induces guided cell migration (Zhu et al., 2019). Moreover, since nerve regeneration is a slow process, electrical stimulation has been introduced as an effective strategy to enhance the axonal growth speed. In Xu et al. (2019), a PLA/PCL microfiber's scaffold, coated with conductive chitosan and polypyrrole (CS/PPy), has been shown to promote, under 100 mV electric stimulation for 2 h every day, the growth and differentiation of PC12 cells and to support the directional growth of neurites. Moreover, Lee J.Y. and co-authors demonstrated the potential use of PLGA-aligned fibers PPy-coated for nerve regeneration, by performing electrical stimulation through two silver-wired electrodes on PC12 cells and rat embryonic hippocampal neurons that showed an improved neurite outgrowth compared to the non-stimulated cells (Lee et al., 2009). More recently, Gisbert Roca F. and co-authors designed PLA–PPy microfibers’ substrates, in which two gold electrodes, placed at the scaffold’s extremes, electrically stimulated Schwann cells (SCs) and the dorsal root ganglia, showing an increase of 19.2% in the maximum length of the axons and an increase of 40% in the area occupied by the axons (Gisbert Roca et al., 2022).

Within the possible application of the aforementioned technologies, a spinal cord injury (SCI) is one of the most prominent. SCs innervate the skeletal muscles and the visceral organs through nerve bundles (Dowlati, 2017), and therefore, the damage of such a nervous complex, for instance, due to traumatic events, could partially or totally impair the muscle sensitivity and functionality. Traumatic SCI is increasingly recognized as a global health priority (Barbiellini Amidei et al., 2022), due to its mortality, morbidity impact, and the requirement of patients’ expensive healthcare system support; the estimated lifetime costs range from $ 1.5 millions to approximately $ 3 millions (Diop et al., 2021). Nevertheless, researchers are working on innovative treatments to restore the motor and vital functions lost. The use of electric and magnetic fields to alter specific targeted neuronal activities, known as neuromodulation (Famm, 2013; Ye et al., 2022), combined with the implantation of biocompatible material inside the lesion to induce neurogenesis, is one of the most attractive. Several studies have highlighted that SCI electric stimulation can recover volitional movements of the upper or lower limbs (Angeli et al., 2015; Angeli et al., 2018; James et al., 2018), and so far, epidural electrical spinal cord stimulation (EES) is the most successful strategy to restore leg motor control in incomplete SCI patients (Wagner et al., 2018). In a recent study carried out by Rowald et al. (2022), a 16-electrode array was surgically implanted on the lesioned area to activate targeted motor neurons with a predefined timing to reproduce the natural spatiotemporal activation pattern during walking. Anyway, EES is limited due to the necessity of a sufficient amount of surviving fibers (Formento et al., 2018); moreover, such a technique does not restore motor autonomy. Alternative strategies able to induce neurogenesis from NSCs, in permissive and guiding biomaterial scaffold environments to overcome the low probability of cell survival, are currently being investigated. The neurogenesis process exploits cell differentiation into the neuronal lineage, in order to generate a tissue bridge that re-innervates the lesioned area (Kumamaru et al., 2019; Damianakis et al., 2022). In this context, an innovative initiative has been proposed within the European project RISEUP^1^ that can combine electric stimulation with neurogenesis to be supported by a biocompatible microfibril's scaffold. The project aims at the regeneration of the injured spinal cord through the development of an implantable electro pulsed biohybrid (EPB) device that supports and guides the differentiation of human induced neural stem cells (iNSCs) combined with mesenchymal stem cells (MSCs) (Caramazza et al., 2021a; Caramazza et al., 2022) through a highly intense and ultrashort pulsed electric field, as intense as those used in electroporation-based technologies (Breton and Mir, 2012; Kotnik et al., 2019; Caramazza et al., 2020a; Caramazza, et al., 2021b; Marracino et al., 2021), and direct current (DC) stimulation protocols to guide cell migration (Dong et al., 2019; Caramazza, et al., 2020b; Naskar et al., 2020), to generate a biohybrid cell bridge on the lesion. From a technological point of view, the EPB device is represented by a set of interdigitated alternating active and ground planar electrodes, to provide DC or pulsed electric fields with 100 µs duration (µsPEFs) stimulation, on which a biocompatible microfibril-based scaffold will be posed to host the target cells. The EPB will use PLA as a scaffold since it is well-known that this polymer is synthetic, biocompatible, biodegradable, and bioabsorbable in the human body and it has been approved by the U.S. Food and Drug Administration (FDA) for nerve regeneration pipelines (Liu et al., 2017; Zhang et al., 2021). Nevertheless, PLA is an insulating material; thus, the microfibrils’ layer could affect the electric field distribution due to the planar electrodes inside the scaffold and could limit cell exposure, compromising the tissue regeneration efficacy.

For this reason, in this paper, we present a numerical study of the PLA microfibrils’ influence on the E-field distribution generated by interdigitated electrodes, to evaluate if the use of such a technology could be compatible with the electric stimulation of cells. The work proposed in this paper is the first and a fundamental step in EPB design that is going to be realized and used in the future for further *in vitro* and *in vivo* investigations planned in the project RISEUP. For these purposes, the reconstruction of realistic microfibrils’ models is needed, and in the following paragraphs, a detailed description of the reconstruction procedure fine-tuned, based on images of the produced microfibrils, will be discussed. The electrical performances of the EPB models obtained with different microfibril distributions are evaluated through numerical simulations. All the results are compared with the electrodes’ device model without microfibrils, to assess the differences with the nominal E-field and to obtain a deep awareness of the possible cell exposure scenarios, as a function of the microfibrils’ spatial distribution parameters.

## 2 Materials and methods

### 2.1 Reconstruction procedure and characterization of the microfibril patch 3D models

A reliable and fast reconstruction method was fine-tuned to obtain realistic microfibrils’ patch models, which are characterized by denser or sparser fibrils’ distribution, organized in a single or a multilayer structure, with homogeneous or heterogeneous diameters and are even characterized by curved and crossed fibrils, in order to represent the scaffold’s imperfections due to the manufacturing process or due to the extracellular fluid (ECM or buffer). Then, 3D microfibril CAD models were placed over the electrodes, oriented perpendicular to the conductors, and inside a hosting ECM in order to obtain the complete EPB device model. The microfibril models were reconstructed starting from the images of real PLA microfibril samples. In particular, the samples used consisted of PLA microfiber lanes obtained by grouping PLA microfibers with a diameter of 10 µm (AITEX, Spain). In order to maintain the alignment of the lane-shaped microfiber bundles, they were heat-sealed together at the samples’ extremities. The PLA microfiber lane was also heat-sealed to the extremities of a PLA film, where the electrodes will be placed. This PLA film was obtained by the casting technique. First, PLA (1 wt%, Goodfellow, ME34-GL-000110) was dissolved in chloroform (Scharlab S.L., CL02032500) and stirred overnight at room temperature. Then, 120 g of the solution was casted into a glass Petri dish with a diameter of 184 mm. The solution was air dried for 5 days in order to allow the evaporation of chloroform. Finally, the PLA membrane was dried in a desiccator with fixed vacuum at 40°C for 2 days. Cross-section images of the microfibril distribution were obtained by optical microscopy (Nikon ECLIPSE 80i) (see Supplementary Figure S1). The sample was cut at approximately 2–3 mm from the external boundaries and then placed vertically on an adhesive base; in order to see in the microscope objective, microfibril circular sections were cut with a 10× magnitude factor. Further top view images were acquired to investigate a particular microfibril shape, such as curved or crossed microfibrils, within the sample. First, the stereomicroscope (Leica, MZ APO) was employed with low-magnitude factors (10× and 20×) to have an overview of the sample spatial distribution, which generally is more compact and straight at the extremities (near the sealing) than in the center. In the scaffold’s central part, the fibers are freer to move, curve, or cross than in a more peripheric one, where they are more straight and sorted because of the tension applied at the substrate junction. Finally, scanning electron microscopy (SEM, Zeiss, AURIGA Compact) was applied to investigate the samples’ imperfections and singularity deeply. Such a technique allows us to acquire images with a high level of accuracy and magnitude factors higher than a stereomicroscope (100×, 150×, 250×, and 500×) to focus on interesting details. Some of the images are reported in the Supplementary Figure S1.

Thus, starting from the collected images, the reconstruction procedure can be briefly described as follows:

• The microfibrils’ cross-section microscopy images are imported in commercial software AutoCAD2023^2^.
• Then, microfibrils’ circular sections are built through octagons in AutoCAD, which are preferred to circular sections for computational issues (Paffi et al., 2007), in order to reproduce the realistic spatial distribution of the lane. In this step, it is possible to create octagons with the same dimensions or modulate them as a function of the variable cross sections shown in the microscopy images.
• 2D sections are extruded to obtain 3D models that replicate the realistic elongated shape of the fibers, which have a length that is able to cover all the electrodes’ strips. It is possible to curve or cross one or more fibrils, as those shown in SEM top view images in Supplementary Figure S1.
• Finally, all the models built are exported as AutoCAD file.dwg.

**FIGURE 1:**
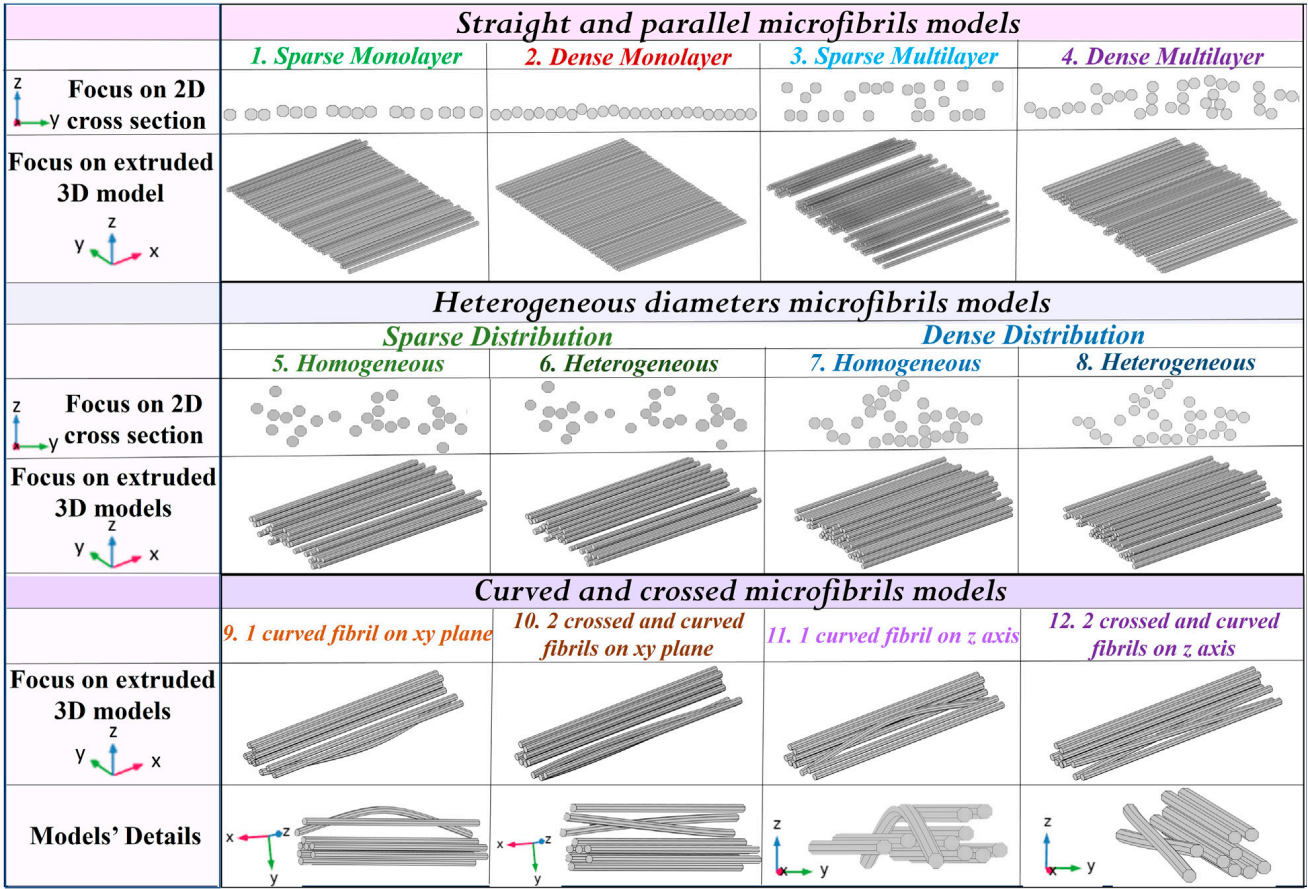
Characterization of different 3D patch models. Straight and parallel microfibril models have been built, considering a sparse and a dense distribution on a single layer or on a multilayer, respectively. Furthermore, other models have been built considering the diameters’ variability and one or more curved or crossed fibrils.

Following the procedure mentioned previously, the first four built patch models consist of straight, parallel, and homogeneous diameter microfibrils in order to investigate the possible effect on the E-field distribution of the lane density, i.e., characterized by a greater or shorter interfibrillar distance. They are all reported in Figure 1 and are defined as a sparse monolayer (model 1) with a height of approximately 25 μm that well-reproduces a still sorted and controlled distribution, but which is closer to the center; and a dense monolayer (model 2), characterized by a height of approximately 10 μm, which is representative of the distribution near the sealing with PLA. The last two patches are a sparse multilayer (model 3), 50 µm high, and a dense multilayer (model 4), approximately 40 µm high, which represents an overlap between the fibers in the sample’s central areas. The reference cross-section images are reported in Supplementary Figure S2. Moving toward more realistic models, taking into account the possible variability in each microfibril’s diameter, four more models have been considered, in particular, a sparse distribution with a homogeneous (model 5) and heterogeneous radius (model 6), and a dense distribution with a homogeneous (model 7) and heterogeneous radius (model 8). Nonetheless, the diameter heterogeneity could have a maximum variability of 20%, according to Gisbert Roca et al., 2021. Finally, one or two curved and/or crossed microfibrils in different planes and orientations have been modeled in AutoCAD, as shown in Figure 1, in a 3D view: a curved fibril (models 9 and 11) and two crossed and curved fibrils (model 10 and model 12) on the transversal xy and longitudinal xz planes (along the *z*-axis) have been added, respectively. Such a realistic spatial behavior has been analyzed from SEM images from a top view (Supplementary Figure S2), which shows that it is possible to find one or more not parallel and sorted fibers, due to fabrication imperfections, in the scaffold’s center. The replication of abnormal fibrils allows us to perform an even more precise dosimetry.

### 2.2 Fine-tuning of the complete scaffolded electrodes’ 3D models

**FIGURE 2:**
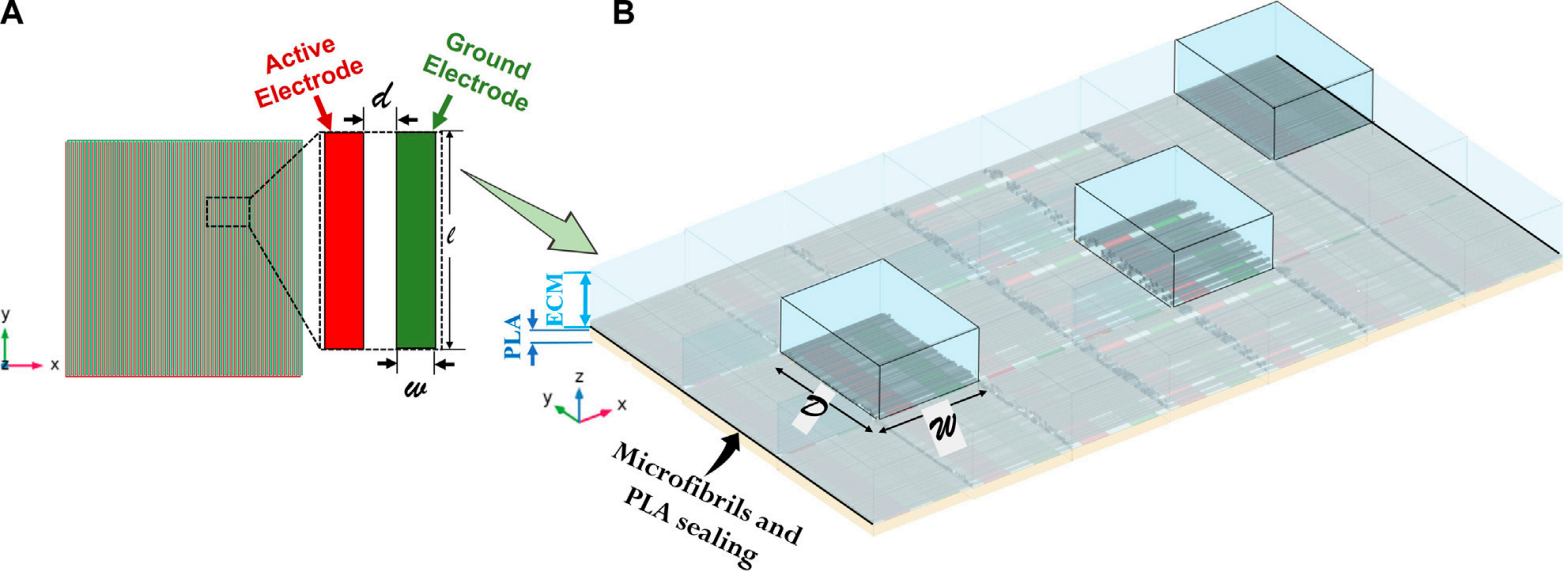
Rationale of the microfibrils’ patch building. **(A)** The electrodes’ technology involves utilizing alternating active and ground planar and interdigitated golden tracks electrodes. **(B)** A couple of electrodes is placed between a PLA substrate and an extracellular medium (ECM), on which continuity periodic conditions are applied to replicate the solution. The microfibrils’ lane is then added on the electrodes’ surface. Each patch is representative of a specific region inside the whole device, for instance in the centre or at the substrate junction.

**FIGURE 3:**
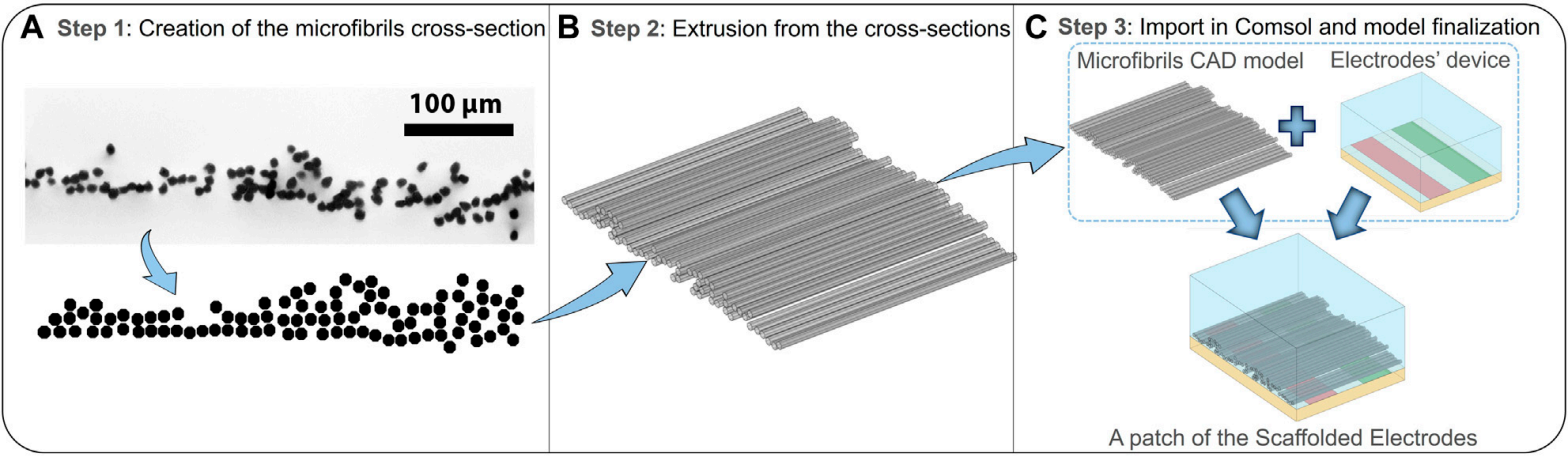
Workflow of the 3D microfibril reconstruction procedure and modeling of the final scaffolded electrodes. **(A)** Step 1: creation of the microfibril octagonal 2D cross section using AutoCAD, starting from optical microscopy images. **(B)** Step 2: extrusion of the cross sections to obtain the final microfibril 3D CAD models. **(C)** Step 3: the import of the built models using Multiphysics software, where the fibers will be scaled and moved to finalize the complete scaffolded electrode model.

**FIGURE 4:**
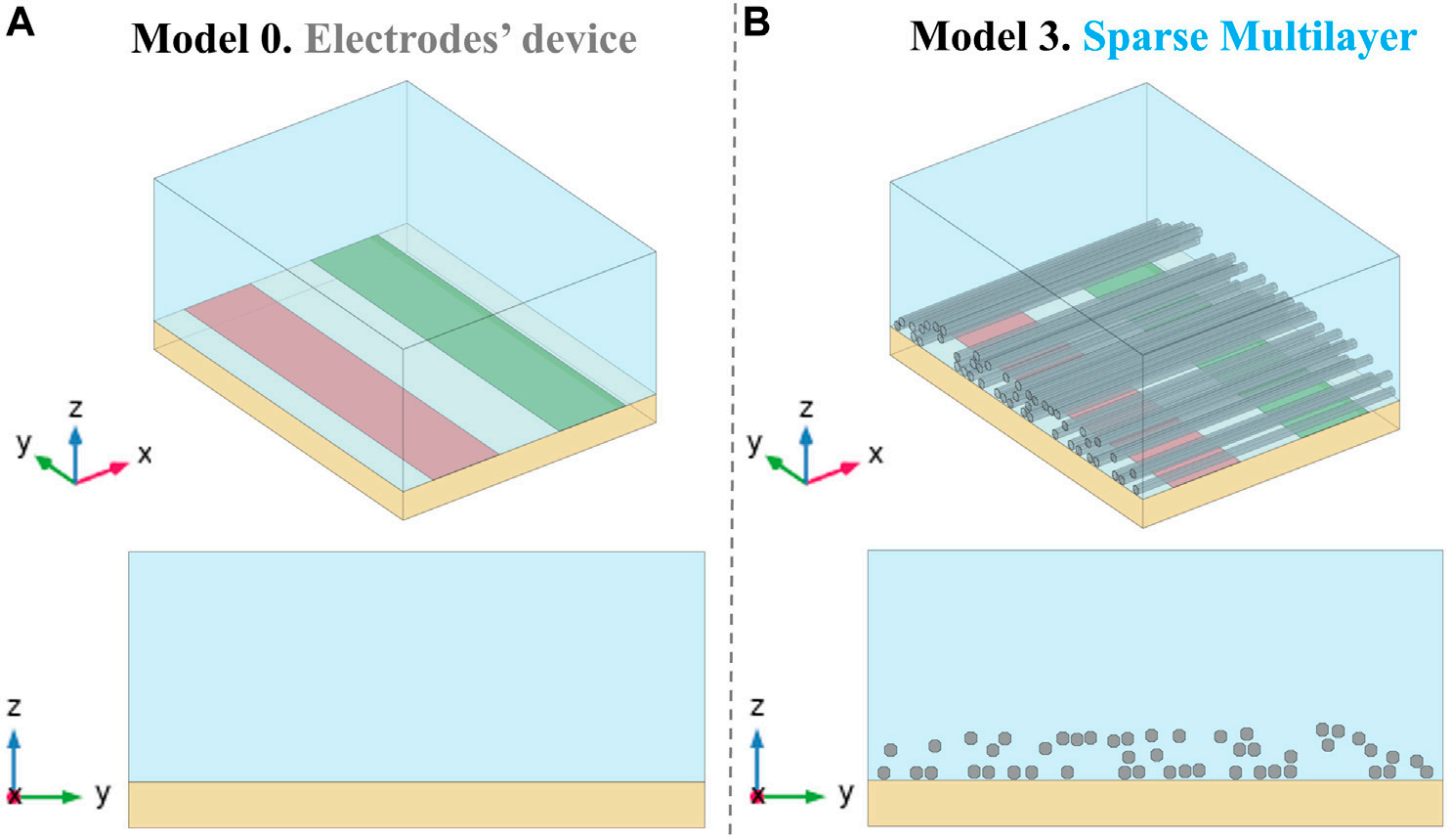
Electrodes’ model placed between the PLA substrate and the ECM volume without a microfibril layer (model 0, Panel **A**) and an example of microfibril positioning inside the electrodes’ device (Panel **B**), on a 3D view and on the cross-sectional zy plane view. The finalized microfibril patch models built are reported in Supplementary Figure S3.

As a first step, the electrodes’ device has been modeled in COMSOL Multiphysics (v. 5.5), involving a block representing the PLA substrate, on which the conductors without thickness (Figure 2) are placed, and an upper block of the extracellular medium. The use of electrodes’ models without thickness (as shown in Panel A of Figure 2) has been validated through a numerical study using COMSOL, in which a RAM occupation reduction of approximately 6% has been assessed. Such a model (defined as model 0) is the reference electrodes’ device model, useful in obtaining the nominal E-field without fibers; subsequently, all the microfibrils’ 3D CAD models, relative to the different scenarios analyzed, are imported in COMSOL Multiphysics, which solves the electro-magnetic problem using the finite element method (FEM) (Jin-Ming, 2015). In order to further reduce the computational cost, the whole microfibrils’ sample (with mm dimension) has been divided into smaller patches that are able to reproduce a particular sample area (Panel B of Figure 2), with a lateral dimension W of 381 µm and a longitudinal dimension D of 500 µm. Every microfibril’s patch includes a couple of active and ground conductors, 100 µm wide. The full reconstruction process is summarized in Figure 3: octagon creation to reproduce the microfibrils’ cross section spatial distribution (Panel A), which are then extruded to obtain 3D models (Panel B); the final microfibrils’ model is imported in COMSOL and placed inside the bare electrodes’ model in order to finalize the patch of scaffolded electrodes (Panel C). In Figure 4, an example of finalized scaffolded electrodes of model 3 is reported, whereas all the other models are shown in Supplementary Figure S3. The fibers are oriented perpendicularly to the conductors, thus following the E-field lines. Moreover, the E-field distribution obtained for all the models will be compared with the nominal one of the electrodes’ device model (model 0).

The simulative procedure has been finally setup assigning the electrical properties of the materials, which are selected from the software library for golden electrodes and from the literature for ECM (De Angelis et al., 2020). The assigned microfibril dielectric properties were experimentally measured, and they consist of an electrical conductivity of $\sigma =4.25∙10^{-11}$ S/m and a relative permittivity of $\varepsilon_{r}=6.75+j∙0.038$ . The mesh building rationale aims at optimizing the computational cost and involves dividing the buffer volume in two equal parts: the first part is the volume just over the electrodes (100 µm high), in which a custom mesh is set to compute the solution more accurately in the region where the cells are supposed to be exposed; however, in the upper volume of the buffer (100 µm high), a rougher mesh is applied. Moreover, the microfibrils have been discretized through an *ad hoc* customized mesh, to guarantee an accurate solution and, at the same time, to avoid exacerbating the computational cost. The physics used is the electric current (ec), with a monopolar pulsed signal of +10 V intensity applied on the active conductor with a duration of 100 µs and a voltage of 0 V on the ground conductor. Finally, all the results have been post-processed using MATLAB (v. 2021B).

## 3 Results

**FIGURE 5:**
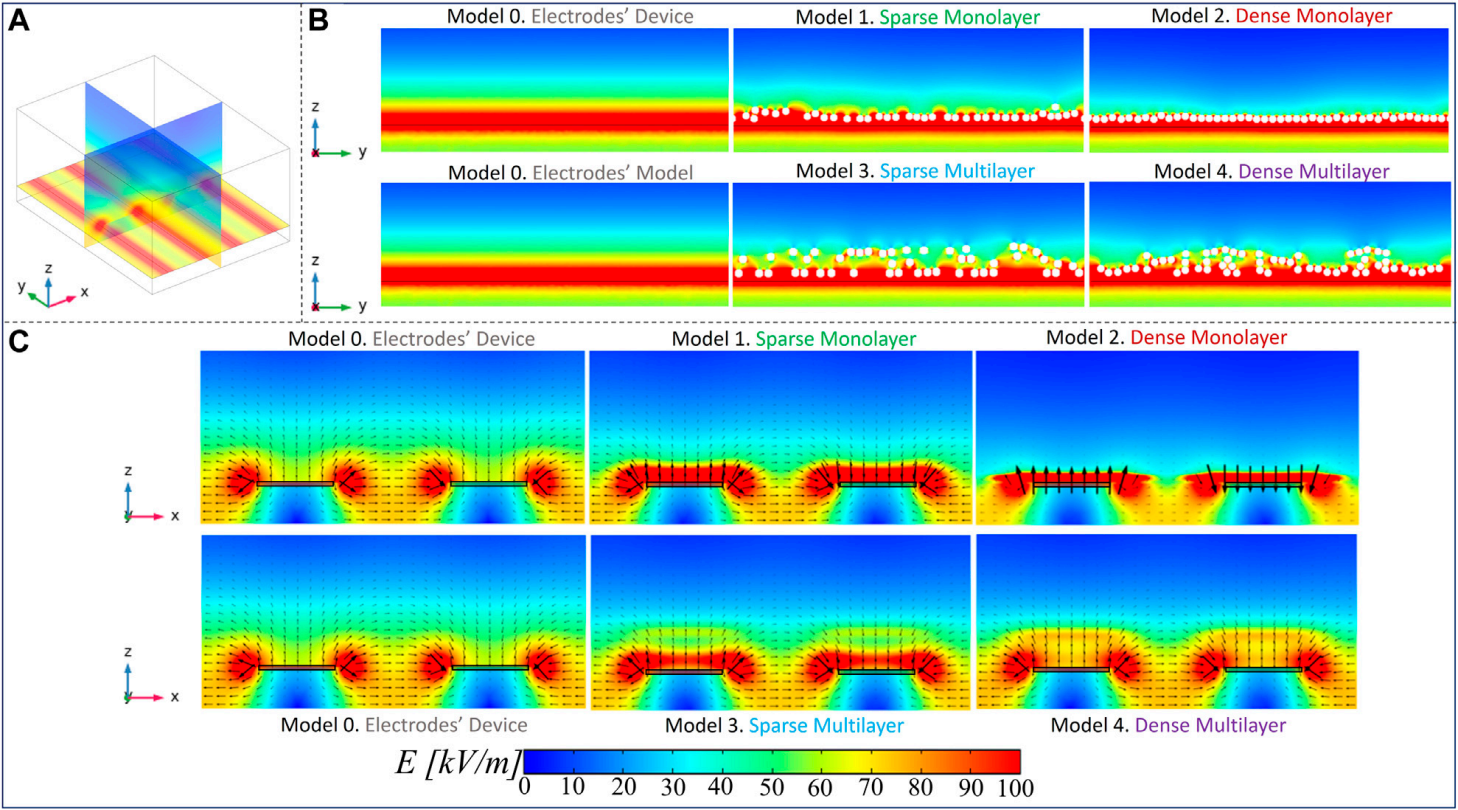
Electric field maps: **(A)** an overview of the E-field distribution in model 0 through a multislice representation on the three coordinated axes; **(B)** E-field distribution comparison between all the models considering the slice zy at the active electrode’s edge; **(C)** E-field maps and field arrows on the zx plane in slices inside the space between the fibrils, to highlight the E-field bridging effect.

In the following section, the main results of the numerical simulations are reported. Several distribution parameters have been analyzed, to determine whether they affect the electrical performances of the electrodes’ device and, consequently, the exposure of cells. The influence of the microfibrils’ scaffold density on the E-field distribution is the first parameter of interest, which is possible to examine considering the sample patches characterized by parallel and straight microfibril models (models 1–4), as defined in the previous section. Panel A of Figure 5 shows a 3D view of multislice maps on the three coordinated axes in model 0, to have an overview of the nominal E-field distribution in a range between 0 kV/m and 100 kV/m. The nominal E-field is higher at the conductors’ edges and then decreases in the gap between the active and the ground conductors but also moving away from the conductors’ plane (z = 0 µm) along the *z*-axis. The reference results of model 0 are compared with those of the microfibrils’ patch models on the active electrode’s edge on the zy axis (Panel B), in which the field inside the fibers is not considered. Here, it is possible to observe that the E-field values induced in the microfibrils’ models, as expected, are slightly lower over the microfibril lane than that in model 0. Thus, the microfibrils’ layer partly shields the E-field, but at the same time, focuses the field in the space between the fibers, bringing it up in the buffer. In relation to the different microfibrils’ density in the analyzed sample’s parts, such an effect is especially emphasized in the sparser regions, where the distance between the fibrils guides the focalization of the field streamlines, allowing the E-field to reach values of up to 100 kV/m.

**FIGURE 6:**
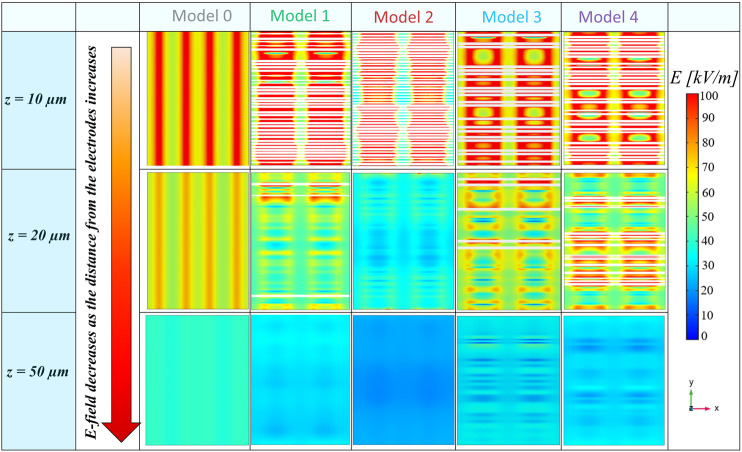
Electric field maps on the xy plane at different heights. The E-field values decrease moving away from the plane of the electrodes (z = 0 µm); moreover, the E-field is focalized in interfibrillar spaces, where the E-field reaches higher values than the nominal one.

**FIGURE 7:**
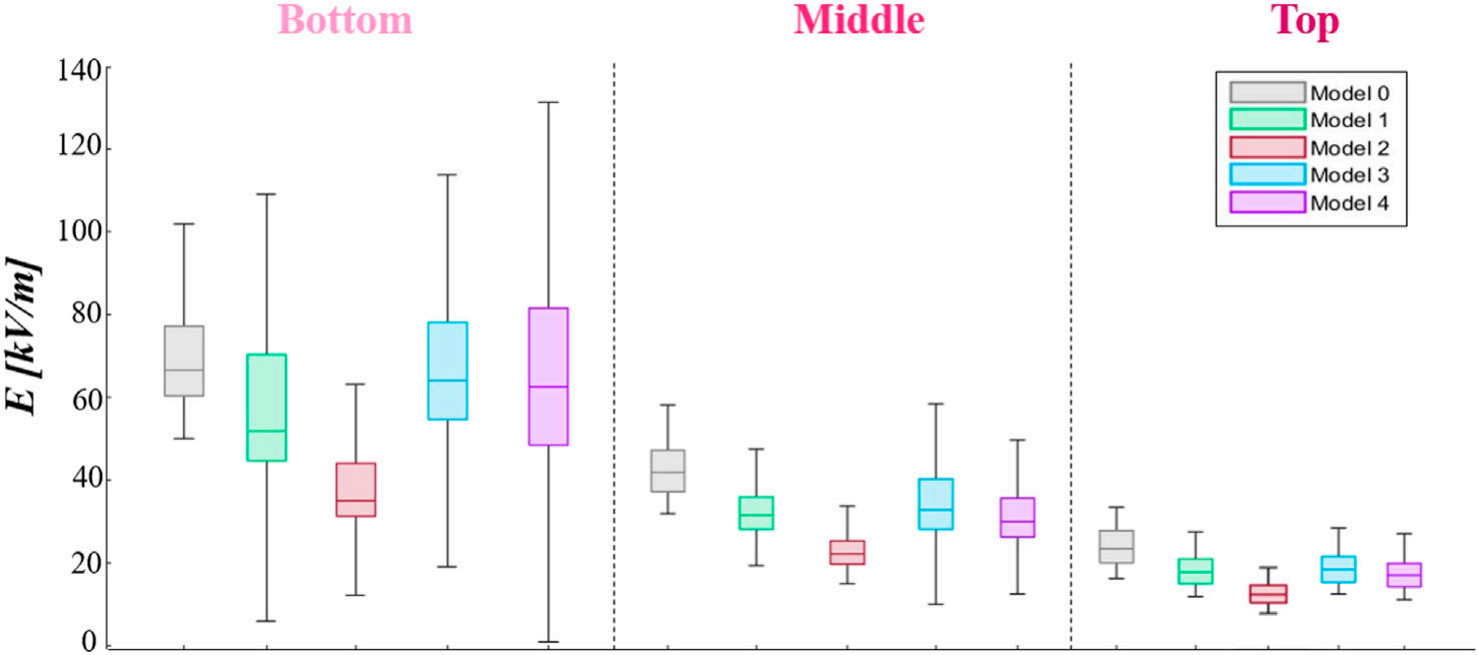
Electric field boxplots from model 1 to model 4, compared with the electrodes’ model (model 0), in three different buffer volumes: in the first 30 µm over the electrodes (0 µm), from 30 µm to 60 μm, and from 60 μm to 100 µm.

These considerations are further confirmed through the results on the zx planes taken in the space between the fibers (Panel C), on which the E-field arrows are reported (a focus of the aforementioned maps is reported in the Supplementary Figure S4, S5, for zy and zx planes). The presence of the fibrils plays an important role in the redistribution of the E-field. In microfibrils’ models, the E-field values over the strips of the electrodes, which are a minimum of 100 kV/m, are higher than that in model 0, on which there is a value of approximately 60 kV/m. Moreover, it is possible to highlight the E-field “bridge effect,” especially inside the multilayer models 3 and 4, where E-field values of approximately 65 kV/m and 80 kV/m are reached in quotes at which an intensity of 45 kV/m is registered in model 0, respectively. The arrows over such maps represent the E-field line direction, and their length is proportional to the E-field intensity, which confirms what was stated previously. A further important consideration is related to the results of the dense monolayer (model 2), which is the model with the most evident reduction of E-field values over the microfibrils’ layer since the fibrils are close to each other, shielding the E-field and reducing the focalization effect. Finally, in Figure 6, we reported the E-field maps on the transversal xy plane, at different heights from the electrodes’ plane (z = 0 µm). In the following maps, the microfibrils are hidden from E-field map visualization and they are represented, wherever present, as white bands. As expected, the E-field decreases, moving away from the electrodes’ plane, and higher E-field values are reached in the interfibrillar space and a major decrease is present in the dense monolayer over the *z*-axis, which is well-shown at z = 50 µm. Furthermore, some areas with fiber-like shapes characterized by reduced E-field values (in a range between 10 kV/m and 20 kV/m, represented in dark blue and blue) are present just above the fibers, as a consequence of the PLA insulating behavior. The aforementioned results have been further investigated, carrying out a quantitative analysis, reported in Figure 7, through E-field boxplots in three different buffer regions (represented from the lightest to the darkest pink shades in Supplementary Figure S6): the bottom volume V_bottom_ represents the first 30 µm above the conductors’ plane (i.e., where the cells will be hosted), the second one is the medium volume V_middle_ (from 30 μm to 60 µm), and the third is the higher or top volume V_top_ (from 60 μm to 100 µm). Notably, the results in V_bottom_, and the E-field median values of models 3 and 4 are in line with the nominal one (respectively equal to 66.6 kV/m, 64.0 kV/m, and 62.6 kV/m), as reported in Table 1 (25th and the 75th percentiles are reported in Supplementary Table S1), whereas the model 1 median value has undergone an E-field median value reduction, which is equal to 51.9 kV/m in comparison with the nominal 66.6 kV/m. As expected, the dense monolayer is characterized by the lower E-field median value, of 35.2 kV/m, a 25th percentile of 31.4 kV/m, and a 75th percentile of 44.1 kV/m. As a whole, the boxplots of models 1, 3, and 4 are more dispersed than the boxplot of the model 0, which means more variability of the E-field values. Notably, for the boxplot of V_middle_ and V_top_, it is possible to highlight that the E-field values induced in the sparser models (model 1 and model 3) are slightly higher than those of the denser models (models 2 and 4), confirming that the denser distributions experience a greater E-field reduction at higher quotes. This consideration is also confirmed in Supplementary Figure S7, in which the E-field histograms of models from 0 to 4 in the buffer volume from 100 μm to 200 µm are reported.

**TABLE 1:** Electric field median value of the boxplots reported in Figures 7–9.

Model	E-field [kV/m]		
	Bottom	Middle	Top
0	66.6	43.1	24.3
1	51.9	32.5	18.4
*2*	35.2	23.0	13.0
*3*	64.0	33.8	18.9
*4*	62.6	31.0	17.6
*5*	59.3	33.9	18.9
*6*	61.0	35.5	19.9
7	63.6	30.1	14.1
*8*	66.1	32.3	15.7
*9*	69.3	36.3	19.6
10	69.9	38.0	19.9
11	65.1	34.8	19.3
12	64.7	34.8	19.2

**FIGURE 8:**
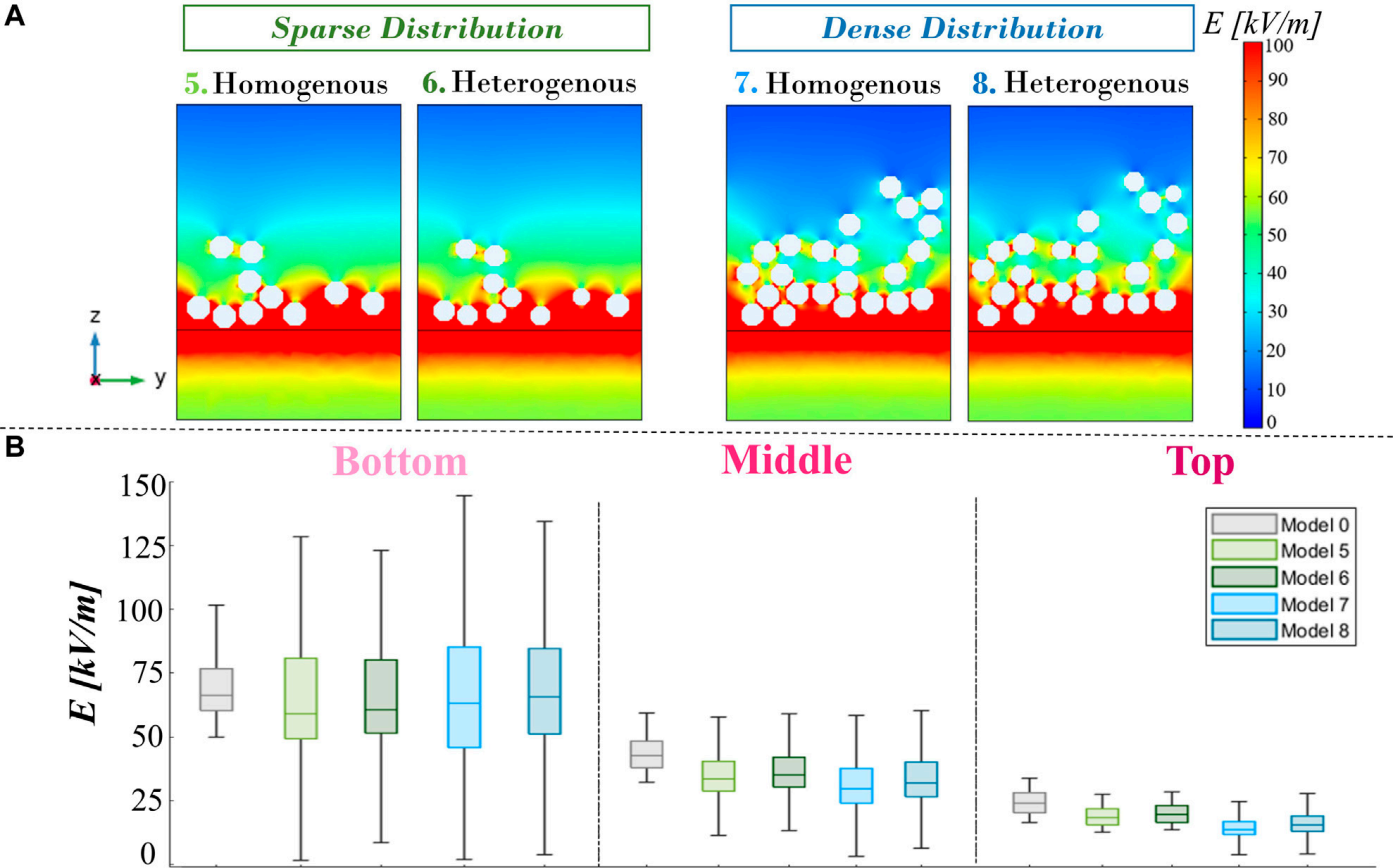
Panel **(A)**: electric field maps for the sparse and dense distribution models with variable diameters, compared with the same distribution but with homogeneous diameters, at the active electrode’s edge on the zy plane. **(B)** Electric field boxplot of the four variable diameter models compared with model 0 in the three buffer regions (bottom, middle, and top).

The second issue investigated is the role of microfibrils’ diameter heterogeneity on the overall E-field distribution inside EPB. For this analysis, we refer to the introduced models from model 5 to model 8 in a sparser and a denser microfibril arrangement, respectively. A focus on the E-field distribution on the zy axis at the electrodes’ edge (Figure 8; Panel A) in patches with heterogeneous diameters is in line with those of homogeneous diameters. These results are confirmed with the boxplots of E-field values in the three buffer volumes (Figure 8; Panel B) (the median values are reported in Table 1). The heterogeneous microfibril patch E-field median, 25th percentile, and 75th percentile values are slightly higher than the homogeneous microfibril patch in V_bottom_, V_middle_, and V_top_. Even if the distribution is denser, the presence of some fibers with a lower radius increases the interfibrillar distance in comparison with the homogeneous patch, favoring the bridge effect and allowing the E-field to pass. For instance, it is possible to highlight that for heterogeneous sparse patches, the E-field median in V_bottom_, V_middle_, and V_top_ increases to approximately 2.9%, 4.7%, and 5.3%, respectively, with respect to the homogeneous patches.

**FIGURE 9:**
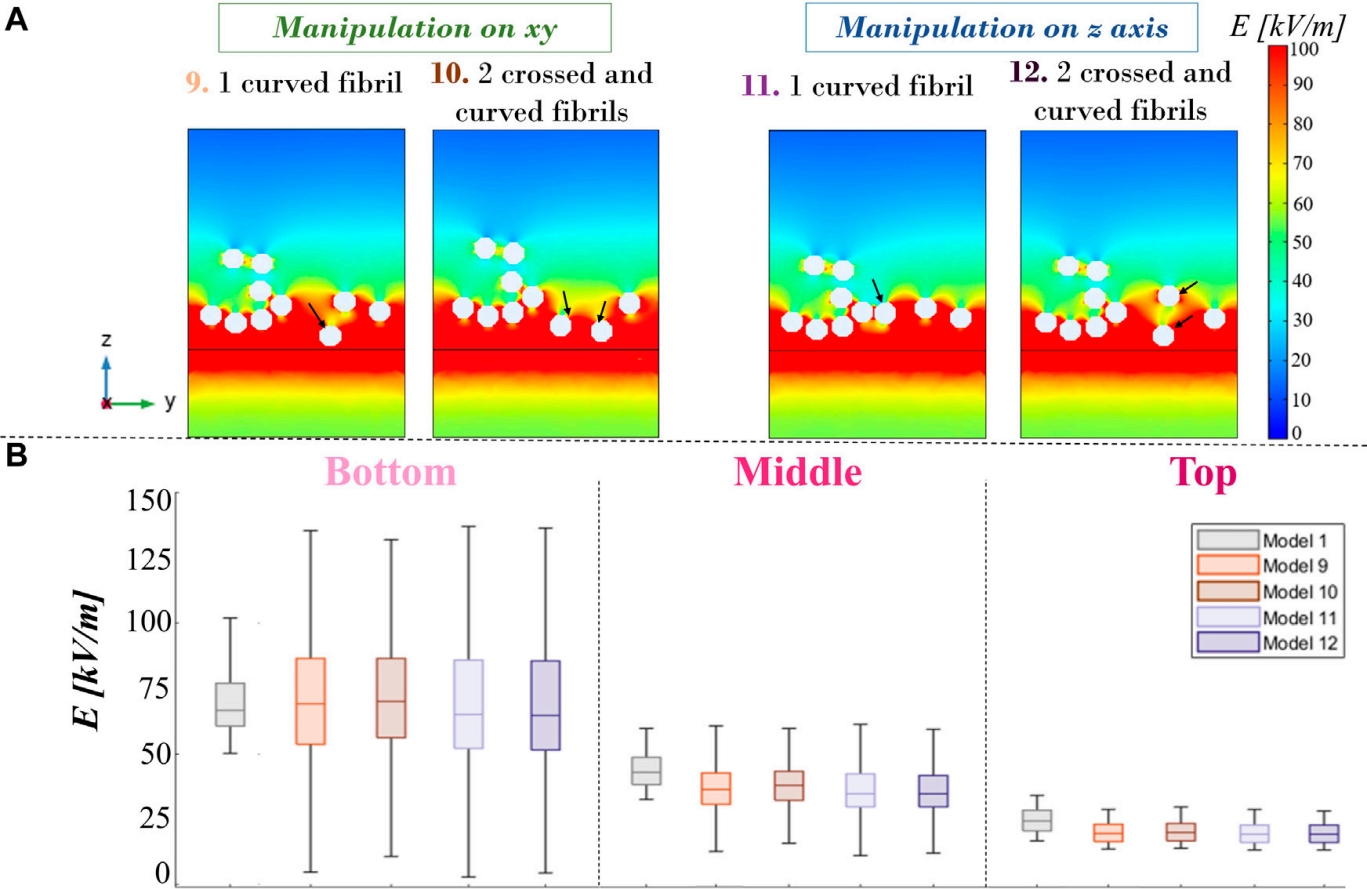
**(A)** Electric field maps for curved and crossed fibrils on a slice at the active electrode’s edge on the zy plane. **(B)** Electric field boxplot of the four models with curved and crossed fibrils compared with model 0, provided for three different buffer volumes.

Finally, concerning models 9 to 12, from maps in Panel A of Figure 9 on the active electrode’s edge, it is possible to see how the E-field distribution changes, as expected, in relation to the inclination and shape of the fibers, indicated with a black arrow. The curvature of the fiber guides E-field focalization and the bridging effect, varying the interfibrillar distance and the height of the layer. Taking into account the boxplots (Panel B), it is possible to affirm that the global statistical analysis shows analog E-field distributions between fiber patch models in the buffer’s volumes of interest. With respect to the nominal field (model 0), in V_bottom_, the E-field median value (Table 1) of models 9 and 10 increases to approximately 4.1% and 5%, respectively, whereas for models 11 and 12, there is a slight decrease of 2.3% and 2.9%, respectively.

## 4 Discussion

In the last few decades, biocompatible scaffolds have been increasingly employed in the field of tissue engineering, particularly for nerve regeneration purposes. In several published works, PLA aligned microfibrils’ structures have been demonstrated not only to be able to create a stable environment to facilitate the cell’s survival but they are also able to promote axonal regeneration in order to create a cell bridge on the lesion and heal the damaged tissue. Although PLA has many advantages, it has an insulating behavior that could be incompatible with the scaffold’s electric functionalization, which is increasingly employed to accelerate the cells’ growth and differentiation processes. For this reason, in this paper, a numerical study on the electric stimulation, provided by planar interdigitated conductors on a PLA substrate, in a microfibrils’ scaffold has been proposed to prove that PLA’s insulating nature does not prevent electric stimulation. For such purposes, a realistic microfibrils’ lane, a dimension of cm order, has been sampled for computational issues in smaller patches, hundreds of µm dimensions, representing the fibers’ spatial behavior in specific regions of the scaffold. Even though the preferred spatial lane configuration provides a sorted and dense monolayer composed of parallel and non-overlapped fibers, it is unavoidable to find modified fibrils, curved, crossed, or agglomerated in two or three layers due to the manufacturing process or in the extracellular fluid presence, especially in the sample’s center. Such realistic distributions have been considered and modeled in 12 different microfibrils’ patches, to obtain a complete analysis of the E-field induced in the scaffolded electrodes in comparison with the nominal field induced in the electrodes’ device without fibers (model 0). Microfibrils’ spatial density, diameters’ heterogeneity, and abnormal shapes (such as curvatures and crossings) are the three main distribution parameters taken into account. From the analysis, the most impacting parameter is the fibers’ spatial density. The microfibrils’ presence focuses the field streamlines in the interfibrillar space, allowing the E-field to pass over the lane and to reach values even over 100 kV/m. The E-field bridging effect plays a key role in buffer functionalization since it can improve cell stimulation in specific regions that can be even higher than the nominal condition. Thus, in the sparse model 1, such an effect is more facilitated than that in the denser model 2, which is representative of a patch near the sealing with the PLA substrate. Consequently, the E-field distribution in all the microfibrils’ patch models is more inhomogeneous as it is possible to see in the boxplots reported in Figure 7, and the E-field intensity over the lane decreases faster than that in model 0 because of the insulating nature of the fibers and also because of the focalization effect in the middle of the lane. Moreover, in denser and overlapped samples’ areas, such as that represented in model 4, in V_bottom_ of the buffer, the E-field median value is in line with the nominal field value (66.6 kV/m); however, moving away along the *z*-axis, it experiences a faster E-field reduction in comparison with the sparser patches. Conversely, taking into account the diameters’ heterogeneity, which is a potential manufacturing imperfection, it is possible to affirm that such a parameter introduces more space between fibers, favoring the bridging effect and E-field penetration inside the buffer more. Finally, the presence of one or more curved or crossed microfibrils also guides the E-field to follow the shape of the fibers, and it is possible to conclude that they do not dramatically affect the E-field distribution, from boxplots in Figure 9.

**FIGURE 10:**
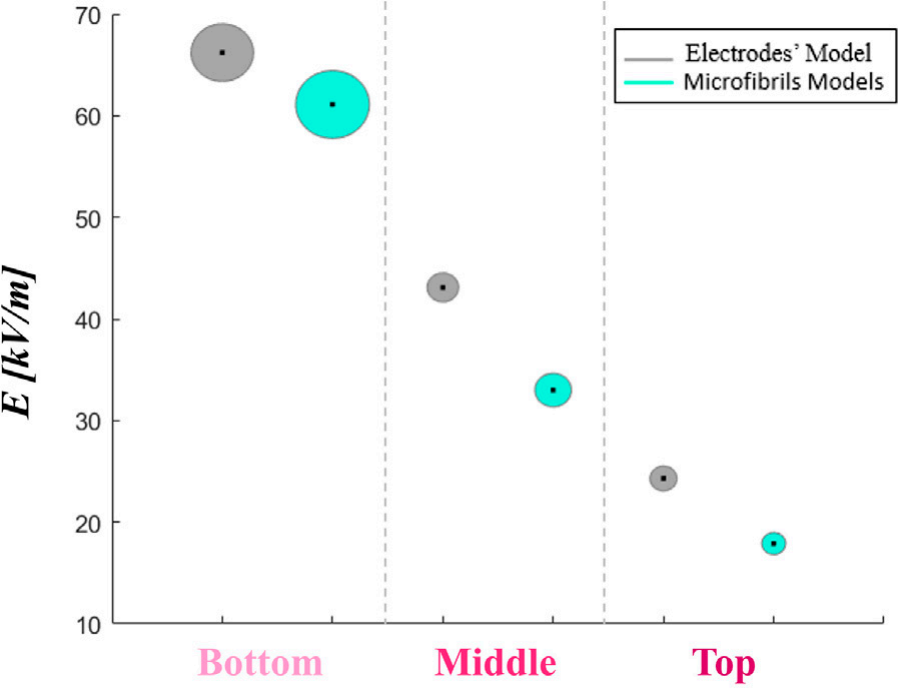
A comparison between the nominal electric field bubble plot of the electrodes’ device and the whole microfibril model averaged values. The bubble area represents the E-field standard deviation, whereas the center point is the E-field median value.

As a final consideration on the microfibrils’ global impact on the E-field distribution, all 12 patches, which have been so far considered singularly through a local analysis, are now pooled together to obtain the averaged E-field values on the whole device in the three buffer regions V_bottom_, V_middle_, and V_top_. These data are compared with model 0 (reported in gray) through a bubble plot representation (Figure 10), where the center of the bubble is the E-field median value and the bubble’s area is the E-field standard deviation. In general, it is worth mentioning that the E-field values obtained in the bare electrodes’ model are in the order of tens of kV/m, as the result of an electric pulse with a 100-µs duration application. In Figure 10, it is possible to notice that the nominal E-field median value is higher in the bare electrodes’ model with respect to the 12 microfibrils’ patches considered as a whole, which are conversely characterized by bigger bubbles than model 0 (i.e., higher standard deviation) in V_bottom_ and V_middle_. However, these interesting results show that in the three buffer regions of the scaffolded electrodes, the E-field is 91.7%, 76.6%, and 73.7% of the nominal one, respectively (Supplementary Table S4). However, the bigger variability of the values is a consequence of E-field redistribution operated by fibrils, in which the bridging effect could be exploited in a possible cell exposure scenario.

### 4.1 Limitations and future work

This study represents a purely numerical investigation into the electrical performance of a fundamental device within the context of the RISEUP project, specifically pertaining to the foundational design of EPB technology and its subsequent development. The primary objective of this work is to lay the groundwork for the realization of the device, by providing valuable insights into how a microfibril distribution may impact the electric field generated by electrode technology and ensure the desired electric field levels. Consequently, this analysis serves as an essential step toward enhancing the manufacturing process’s awareness and efficiency.

Given the inherent challenges in experimentally measuring the electric field produced by this technology, our approach relies on advanced and realistic 3D simulations. These simulations, as proposed here, represent the sole means of determining precise electrical dosages at a microscale level (P. Liu and Miller, 2020; Bai and Gu, 2016; Haider et al., 2021). It is worth noting that, while this study is an integral part of the EPB implementation process, it does not encompass the *in vitro* and *in vivo* validation of its efficacy.

Recognizing the constraints associated with the electrode technology, our investigation focuses on analyzing the absorbed current, estimating values that are in line with the technological limitations. In our future endeavors, we aim to validate the functional efficiency of EPB on the cellular behavior through experimental studies, building upon the key findings discussed in this study. Specifically, both *in vitro* and *in vivo* assays will verify whether cellular stimulation can facilitate tissue regeneration by fostering the creation of a biohybrid cell bridge. This aspect holds pivotal significance in evaluating the technology’s potential applications in spinal cord injury treatment.

To provide valuable insights and predictions concerning threshold levels induced by stimulation, we anticipate conducting additional numerical investigations to complement the experimental *in vitro* data. These investigations will serve as a vital resource in further supporting our research efforts.

## 5 Conclusion

In this work, a reconstruction procedure of microfibrils’ 3D CAD models has been presented, from which a numerical study on the E-field distribution inside an example of scaffolded electrodes has been fine-tuned. From the results shown, it is possible to affirm that this PLA microfibril-based technology, despite its insulating nature, is not only able to support and guide cell growth, but it is also compatible with their electric stimulation, in which approximately 90% of the nominal field is guaranteed in the buffer’s volume hosting the cell. Furthermore, the use of the PLA microfibrils’ scaffold is suitable for the application of high-intensity µsPEF stimulation, and in conclusion, it could be exploited within the RISEUP project.

## Data Availability

The original contributions presented in the study are included in the article/Supplementary Material; further inquiries can be directed to the corresponding author.

## Footnotes

1 RISEUP, RISEUP (riseup-project.eu).

2
https://www.autodesk.com/products.
