# Supplementary material for: Electric field bridging-effect in electrified microfibrils’ scaffolds
Source: Front Bioeng Biotechnol. 2023 Oct 25;11:1264406. doi: 10.3389/fbioe.2023.1264406 (PMC10634785; doi:10.3389/fbioe.2023.1264406)
Supplement: Supplementary file 1 [file Presentation1.pptx]

## Slide 1
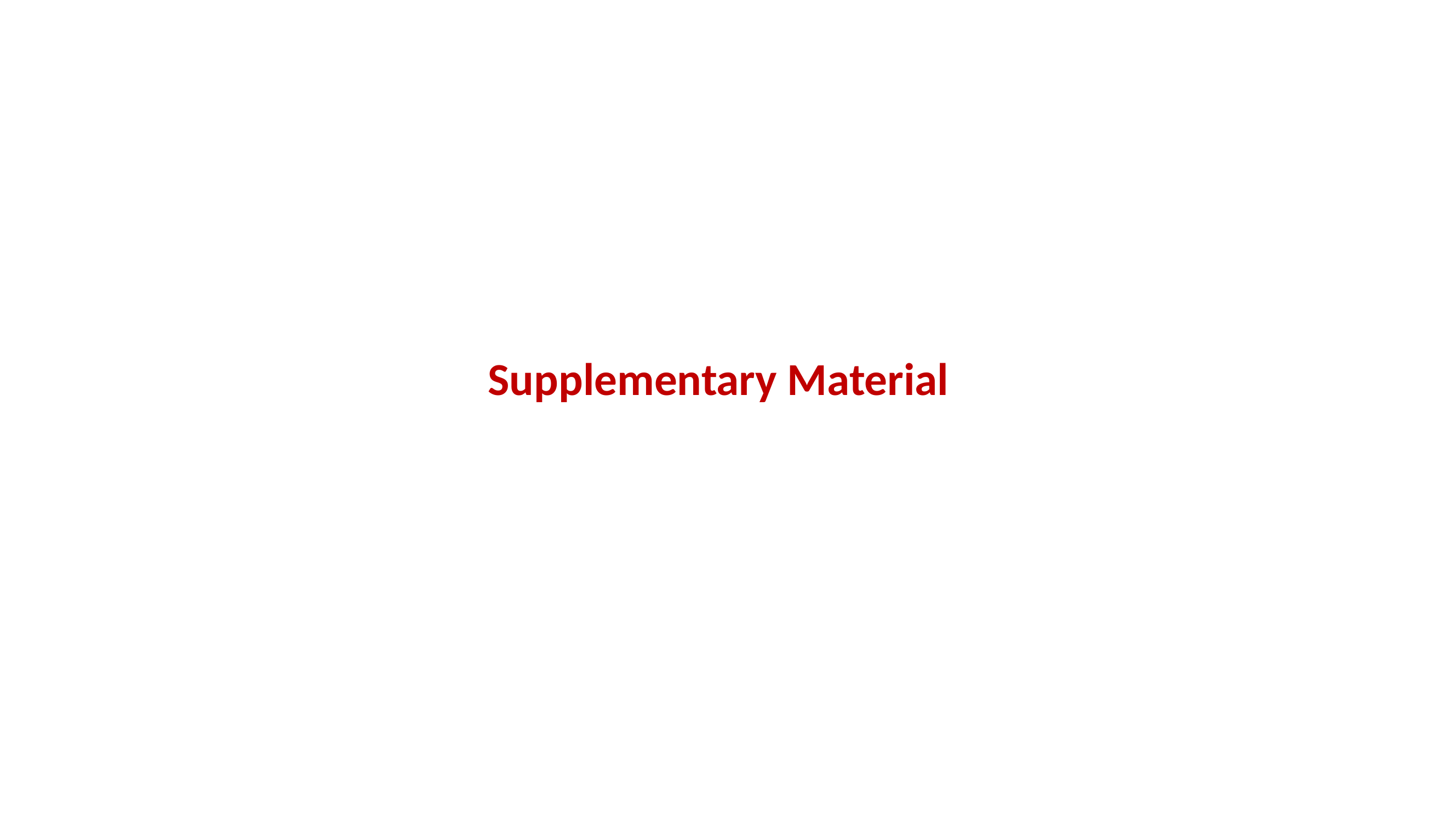

Supplementary Material

## Slide 2
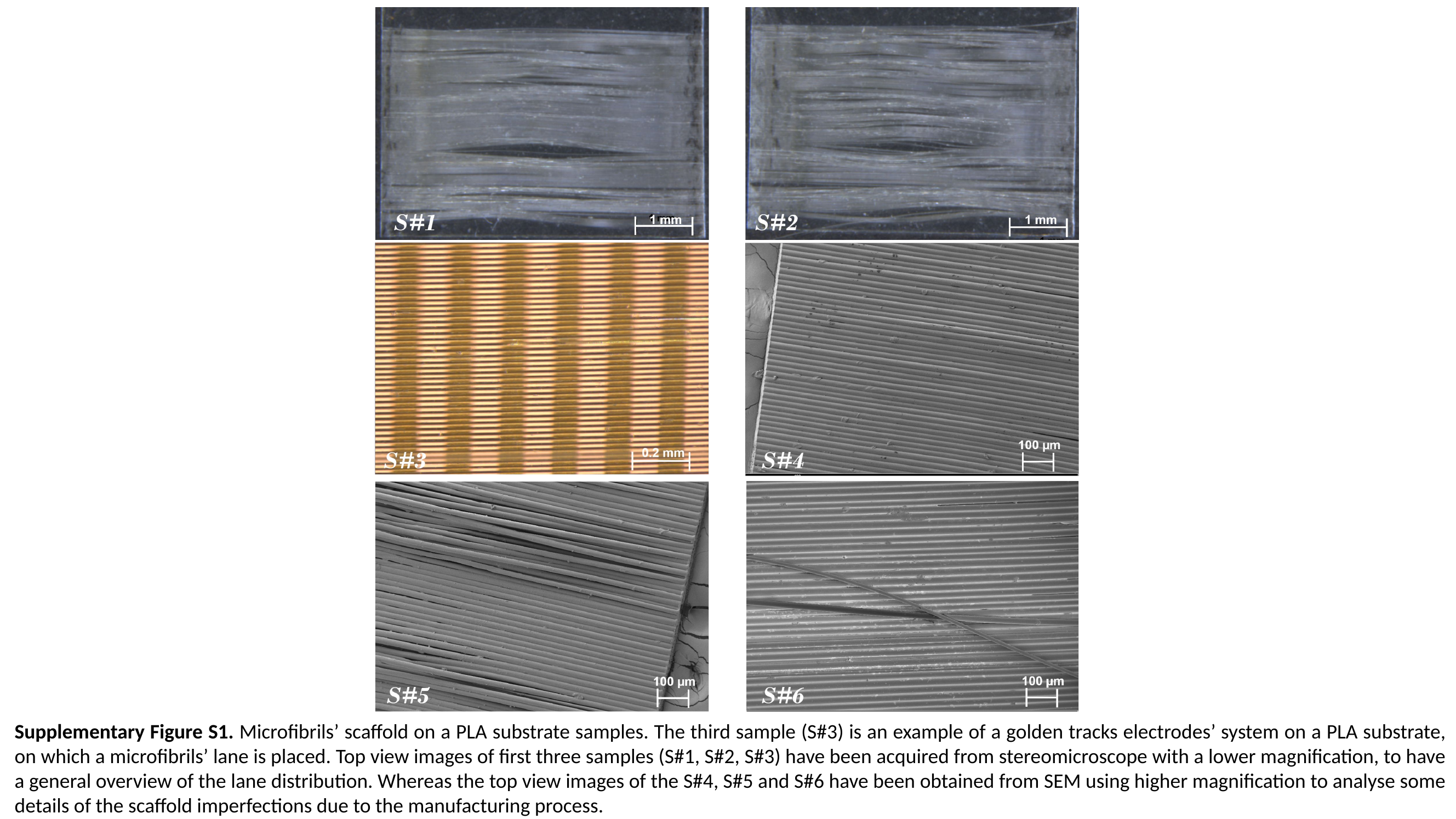

Supplementary Figure S1. Microfibrils’ scaffold on a PLA substrate samples. The third sample (S#3) is an example of a golden tracks electrodes’ system on a PLA substrate, on which a microfibrils’ lane is placed. Top view images of first three samples (S#1, S#2, S#3) have been acquired from stereomicroscope with a lower magnification, to have a general overview of the lane distribution. Whereas the top view images of the S#4, S#5 and S#6 have been obtained from SEM using higher magnification to analyse some details of the scaffold imperfections due to the manufacturing process.

## Slide 3
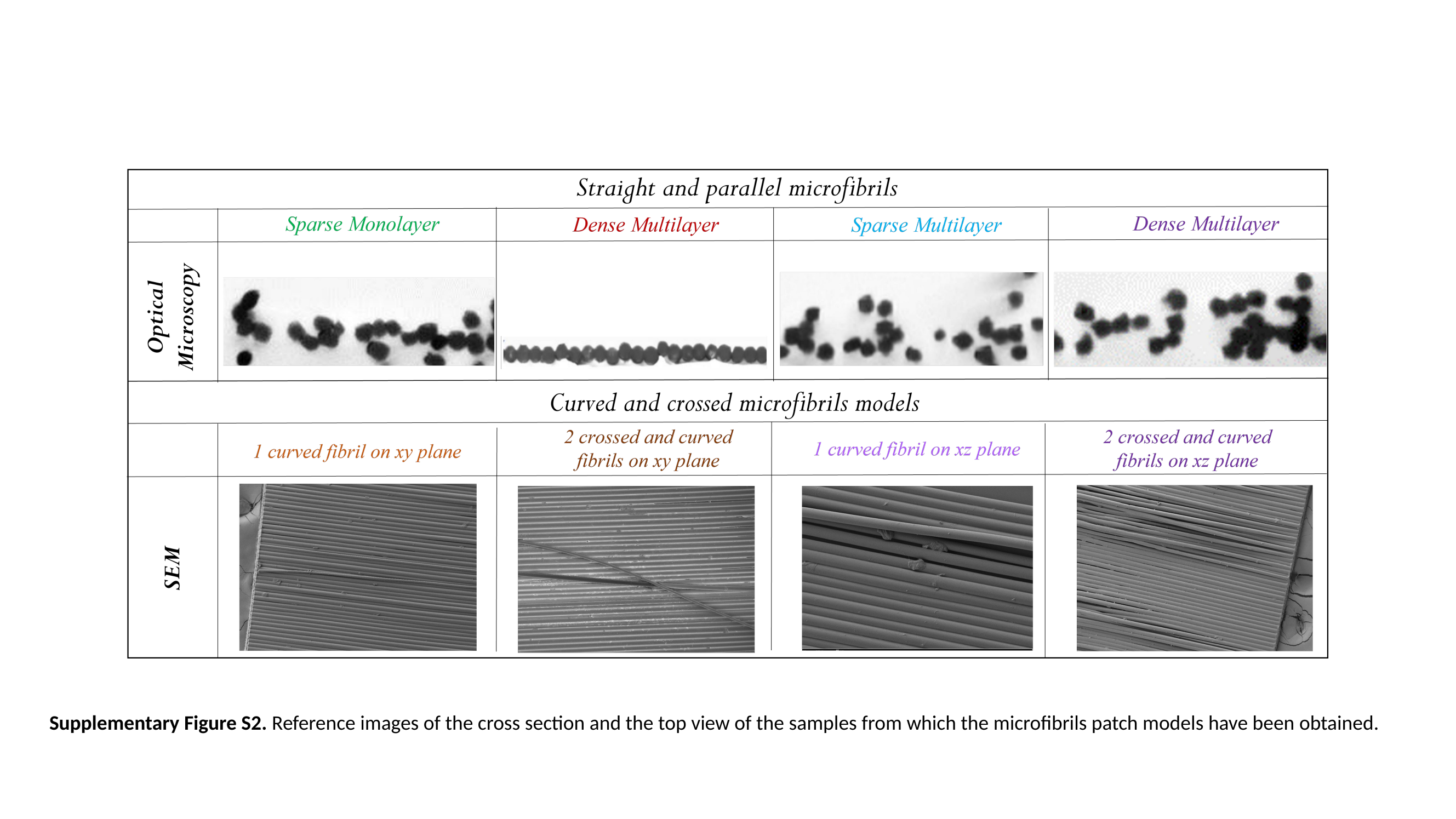

Supplementary Figure S2. Reference images of the cross section and the top view of the samples from which the microfibrils patch models have been obtained.

## Slide 4
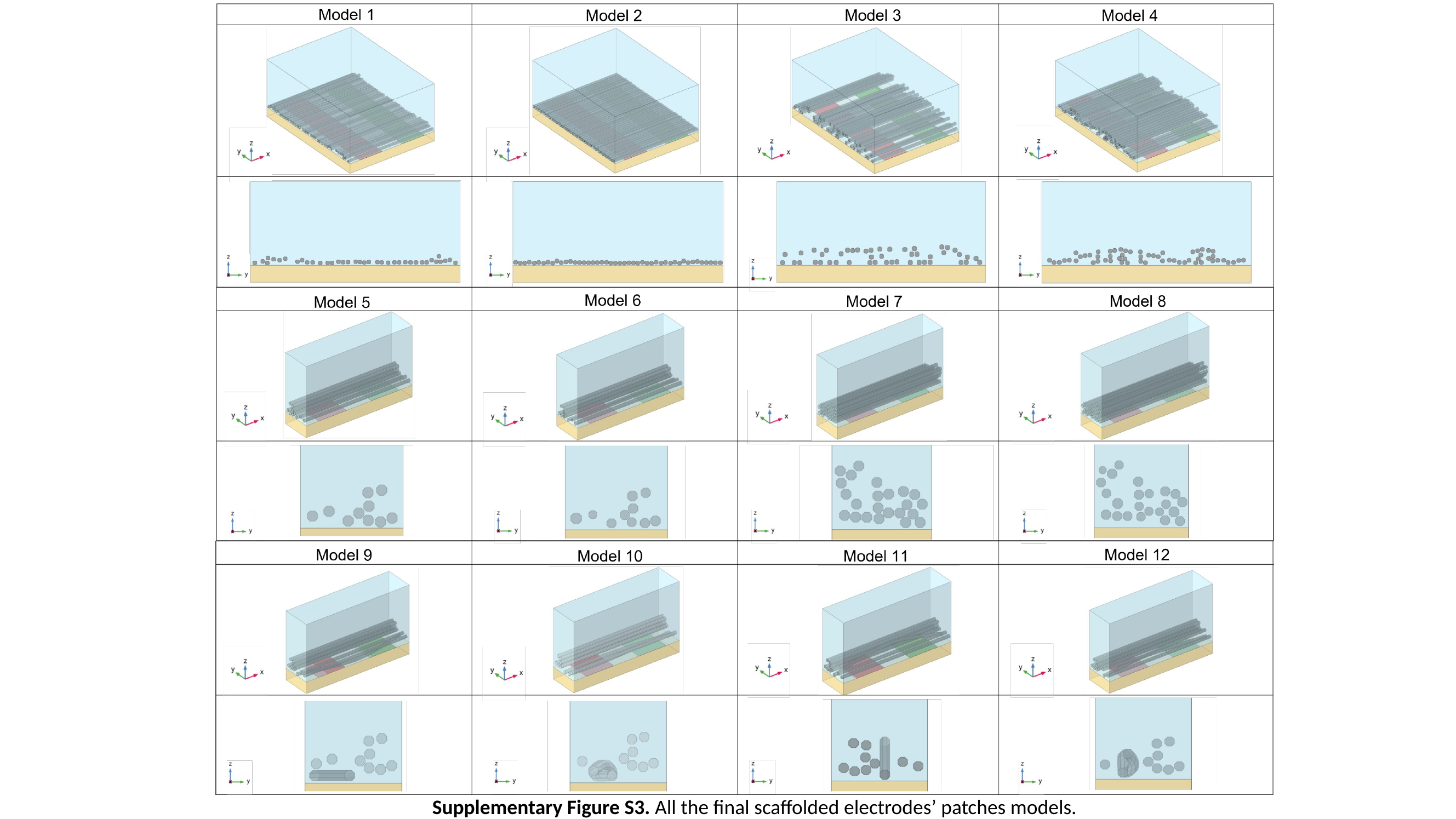

Supplementary Figure S3. All the final scaffolded electrodes’ patches models.

## Slide 5
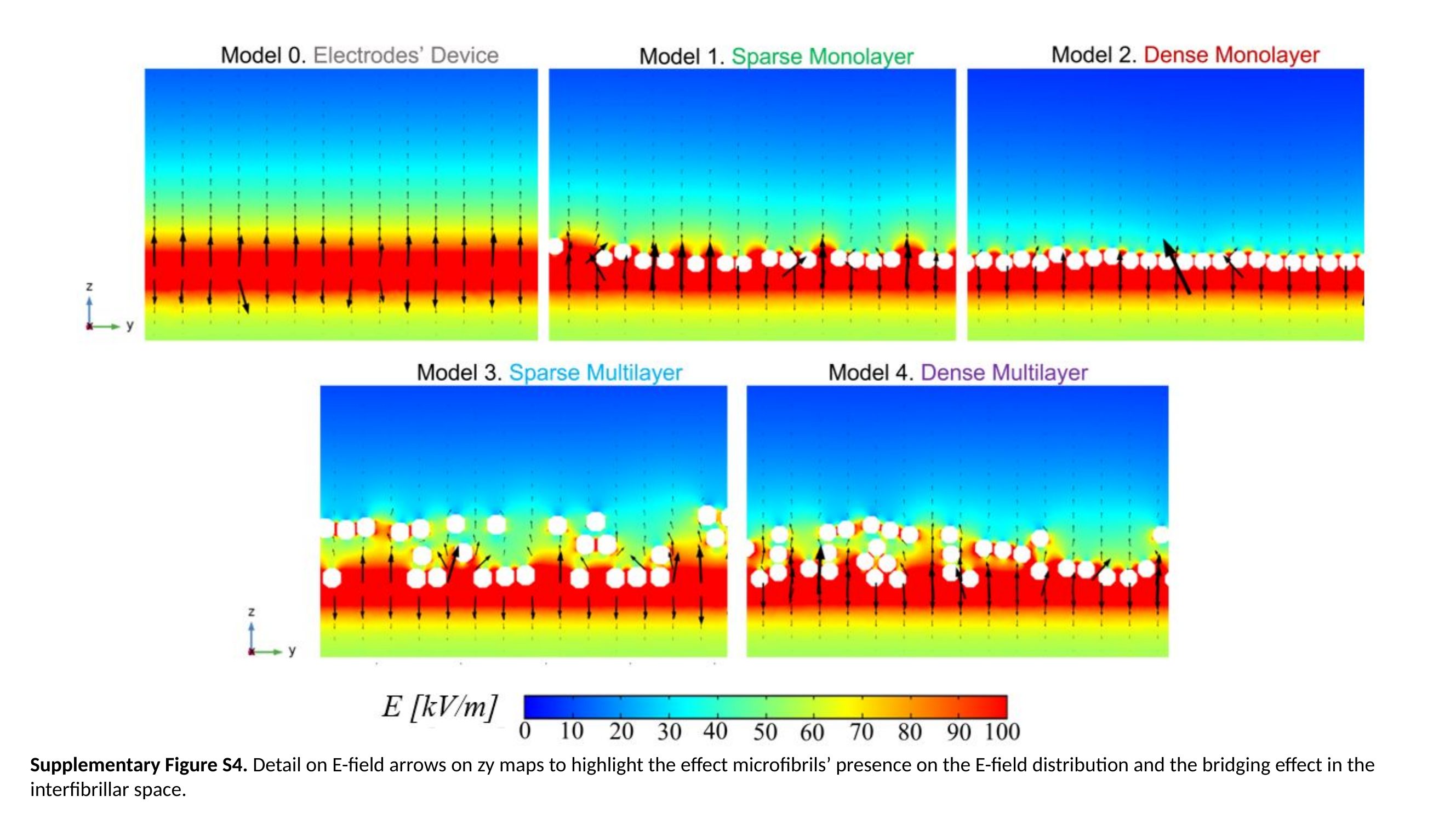

Supplementary Figure S4. Detail on E-field arrows on zy maps to highlight the effect microfibrils’ presence on the E-field distribution and the bridging effect in the interfibrillar space.

## Slide 6
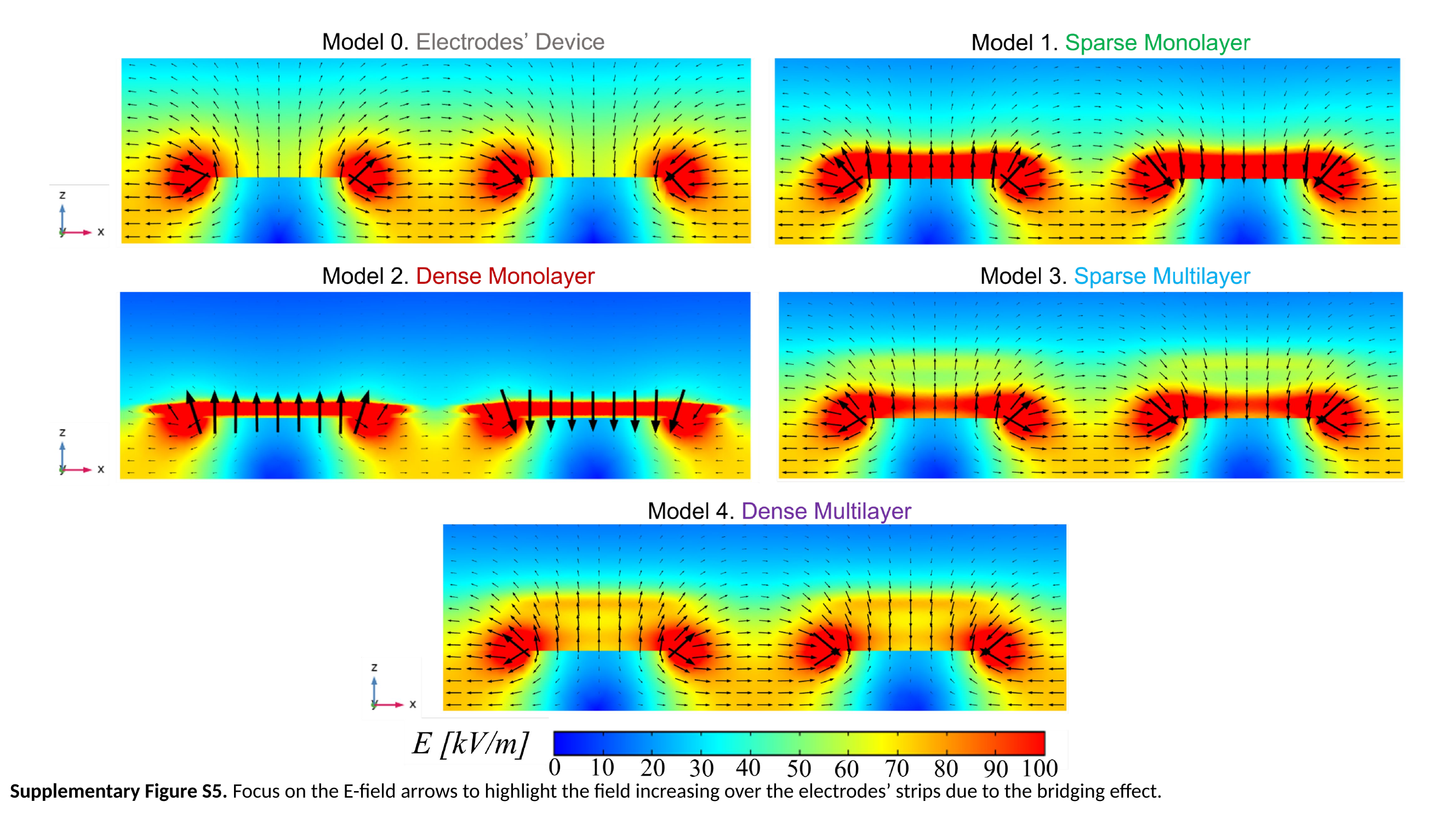

Supplementary Figure S5. Focus on the E-field arrows to highlight the field increasing over the electrodes’ strips due to the bridging effect.

## Slide 7
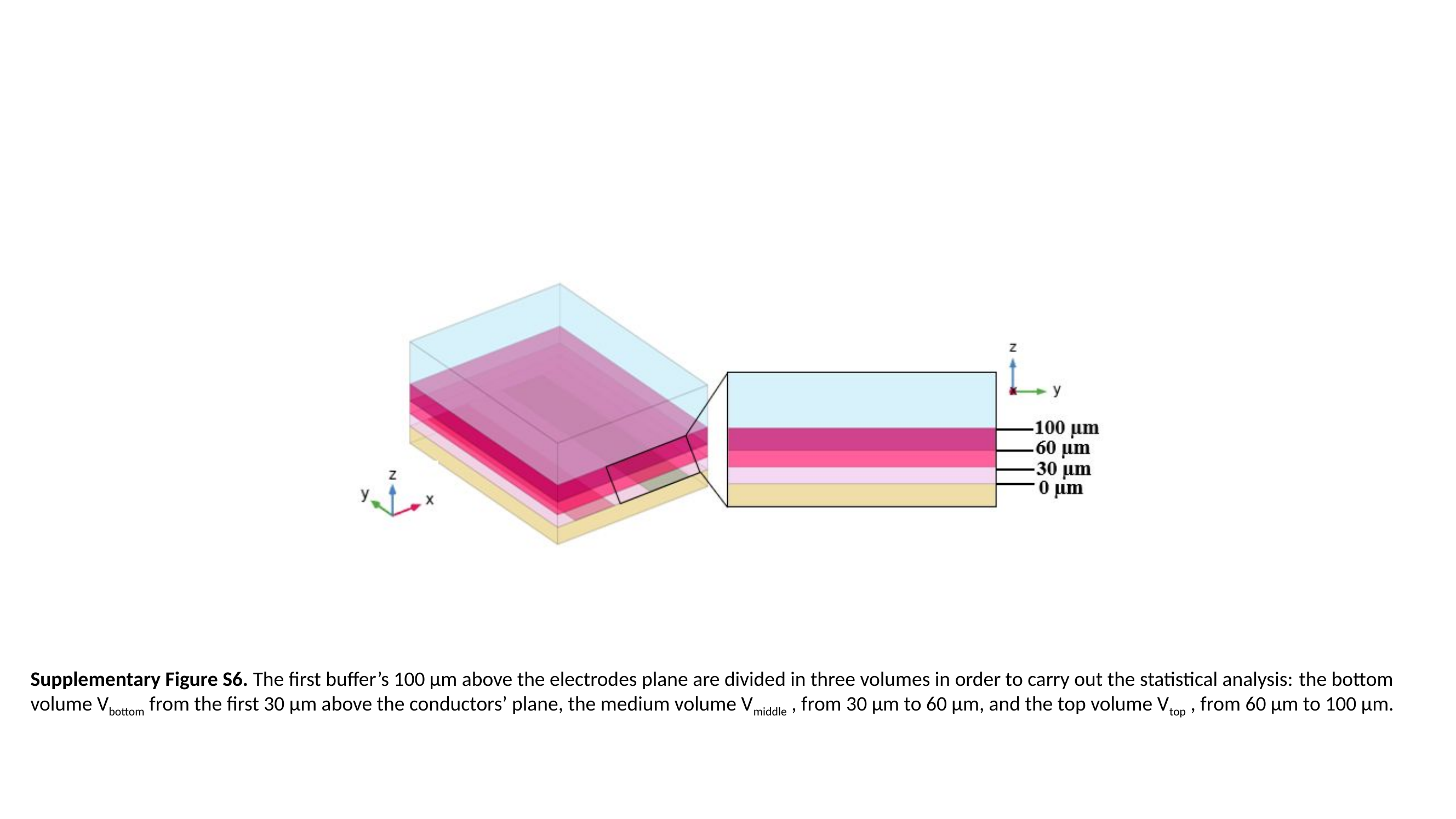

Supplementary Figure S6. The first buffer’s 100 µm above the electrodes plane are divided in three volumes in order to carry out the statistical analysis: the bottom volume Vbottom from the first 30 µm above the conductors’ plane, the medium volume Vmiddle , from 30 µm to 60 µm, and the top volume Vtop , from 60 µm to 100 µm.

## Slide 8
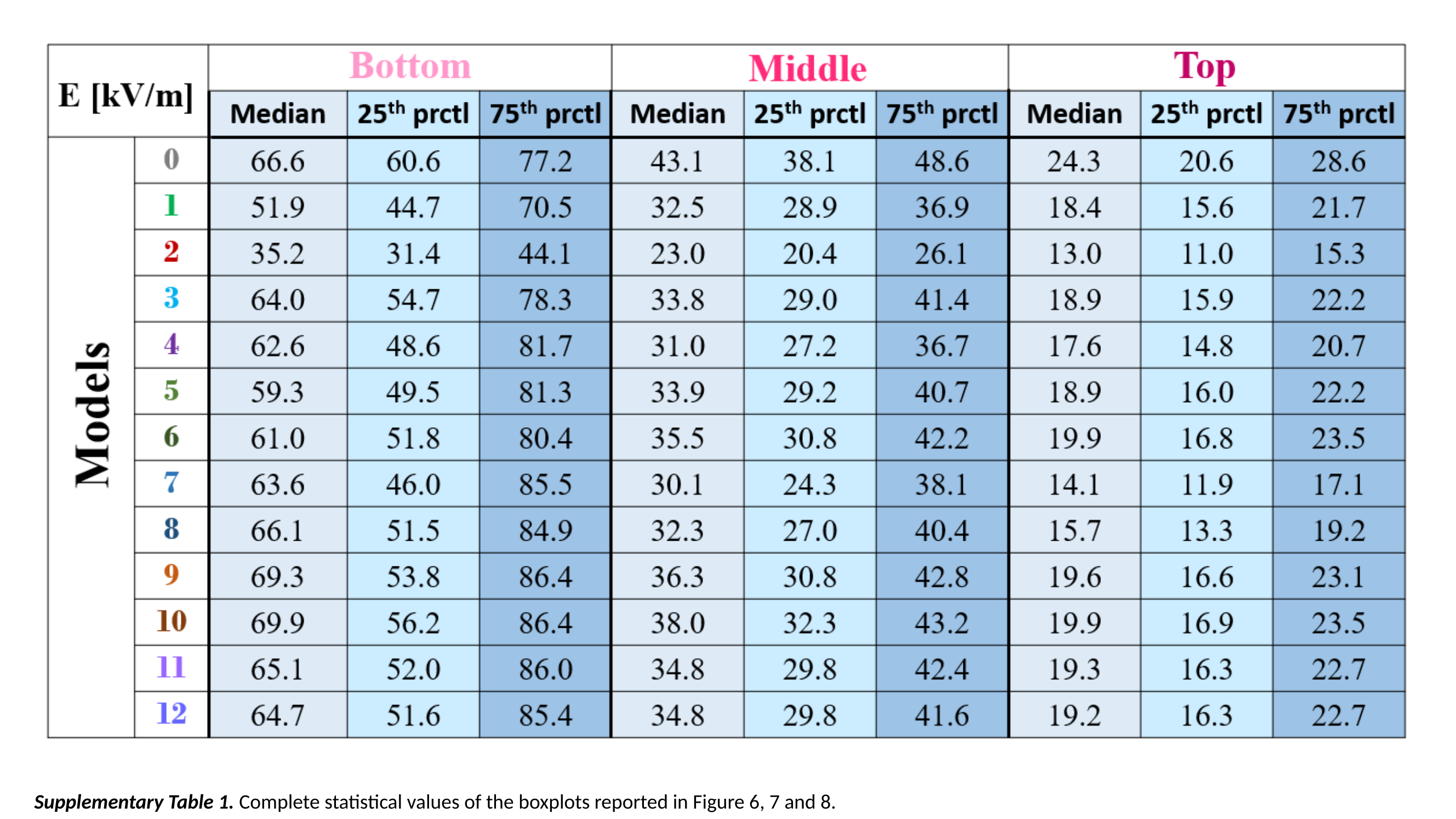

Supplementary Table 1. Complete statistical values of the boxplots reported in Figure 6, 7 and 8.

## Slide 9
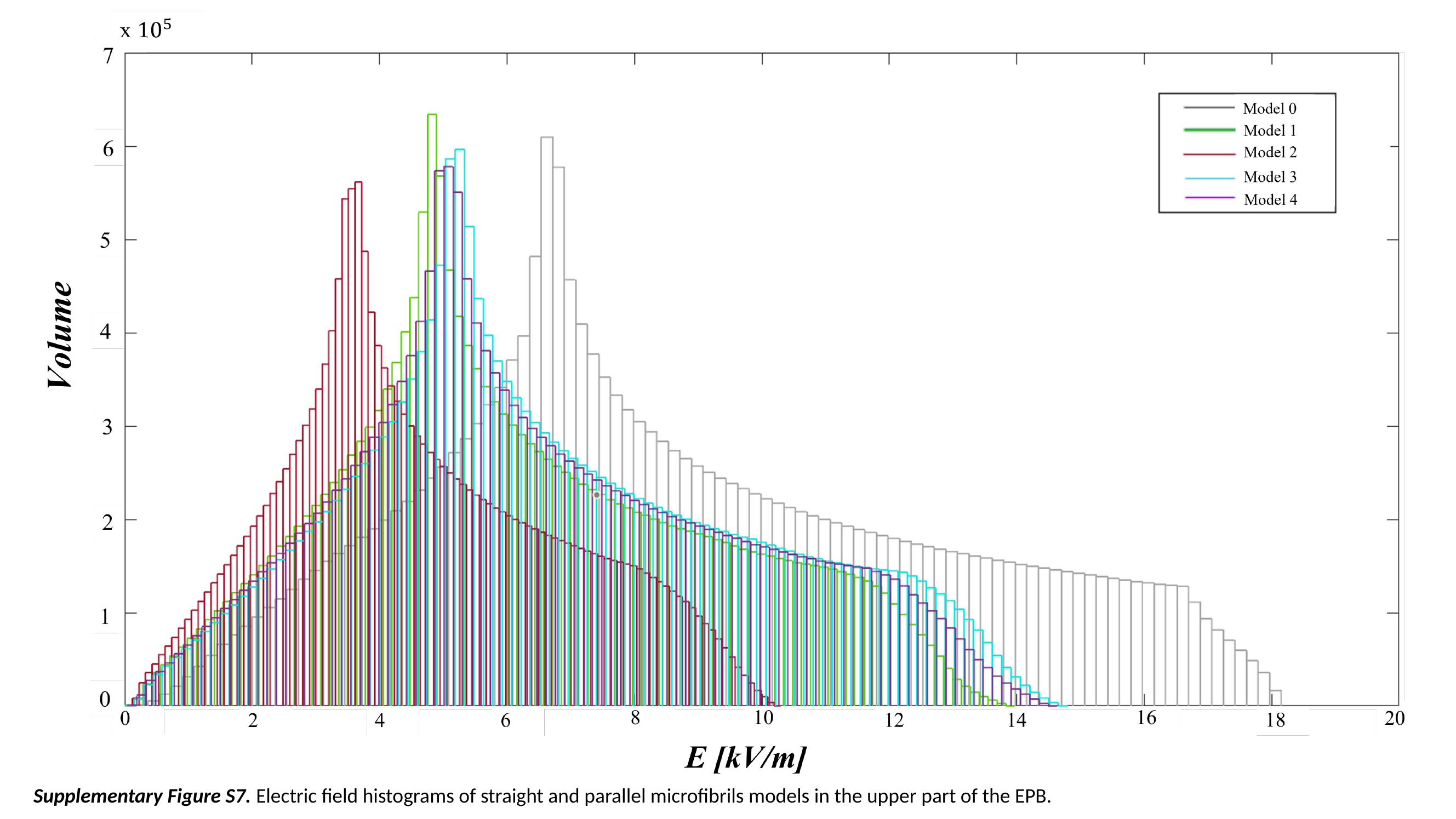

Supplementary Figure S7. Electric field histograms of straight and parallel microfibrils models in the upper part of the EPB.

## Slide 10
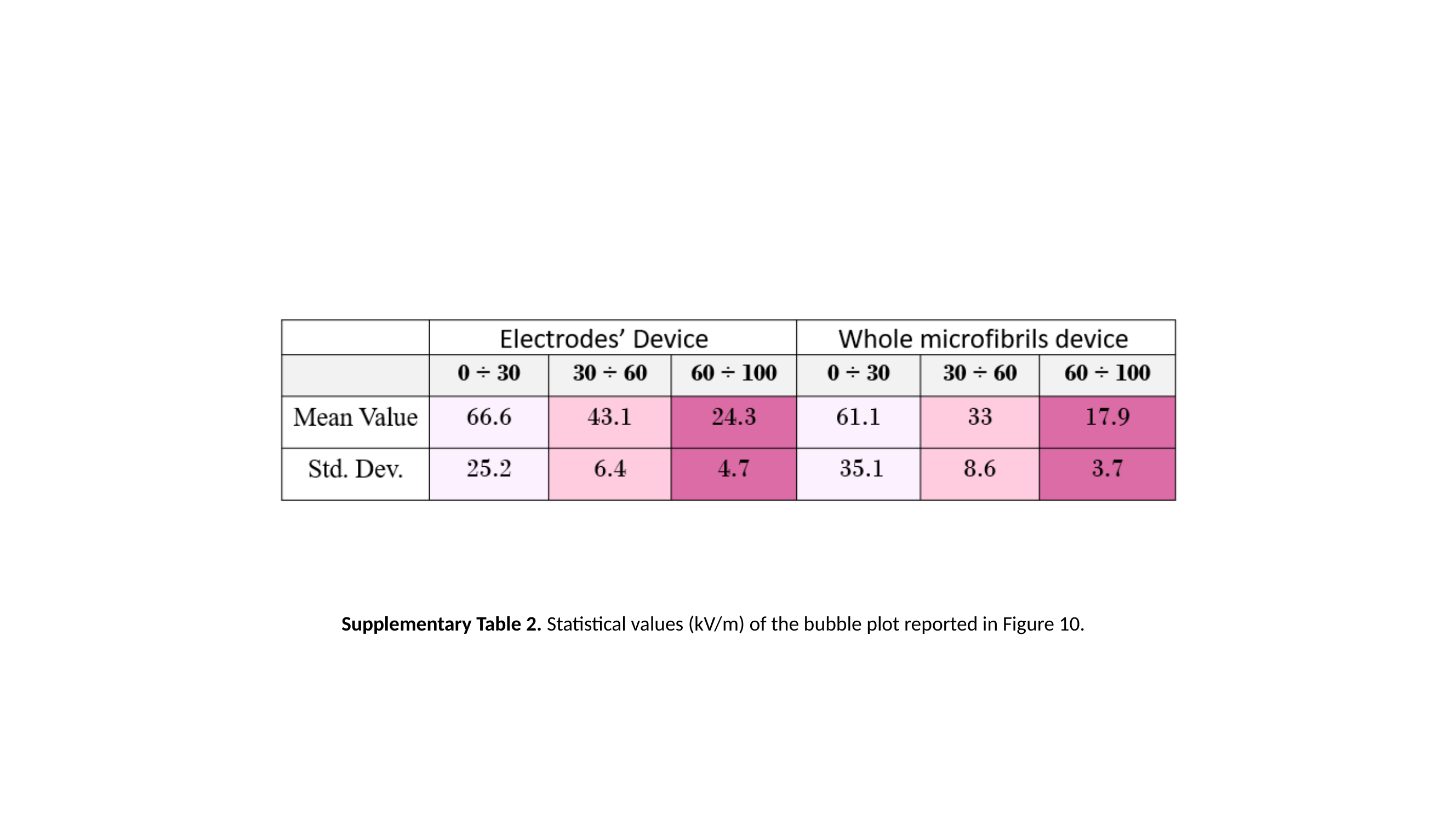

Supplementary Table 2. Statistical values (kV/m) of the bubble plot reported in Figure 10.
